# Pancréatite aigüe du post-partum: à propos d'un cas

**DOI:** 10.11604/pamj.2013.15.2.2359

**Published:** 2013-05-02

**Authors:** Brahim Boukatta, Hicham Sbai, Said Ait Laalim, Iman Toughrai, Nawfel Houari, Abderrahim El Bouazzaoui, Nabil Kanjaa

**Affiliations:** 1 Service de Réanimation polyvalente du CHU Hassan II de Fès Université Sidi Mohammed Ben Abdellah; 2 Service de chirurgie viscérale au CHU Hassan II de Fès, Maroc Université Sidi Mohammed Ben Abdellah

**Keywords:** Pancréatite aigüe, grossesse, lithiases, traitement, acute pancreatitis, pregnancy, lithiasis, treatment

## Abstract

La pancréatite aigüe est une complication rare durant la grossesse. Son incidence est de 1 pour 1000 à 3000 grossesses. L'origine lithiasique est de loin la plus fréquente. Elle survient le plus souvent au cours du 3^ème^ trimestre ou précocement dans le post-partum. Son pronostic dépend de la rapidité du diagnostic. Nous rapportons un cas de pancréatite aigüe grave d'origine biliaire survenant deux semaines après l'accouchement chez une patiente de 24 ans avec une bonne évolution.

## Introduction

La pancréatite aigüe est la plus fréquente des anomalies exocrines du pancréas [1, 2]. Sa survenue durant la grossesse est rare, son incidence est de 1 pour 1000 à 3000 grossesses. Elle survient le plus souvent au cours du 3^ème^ trimestre ou précocement dans le post-partum [3]. Les principales causes sont représentées par les lithiases des voies biliaires et des dyslipidémies [4]. Nous rapportons un cas de pancréatite aigüe d'origine biliaire, survenant deux semaines après l'accouchement avec bonne évolution.

## Patient et observation

**Figure 1:**
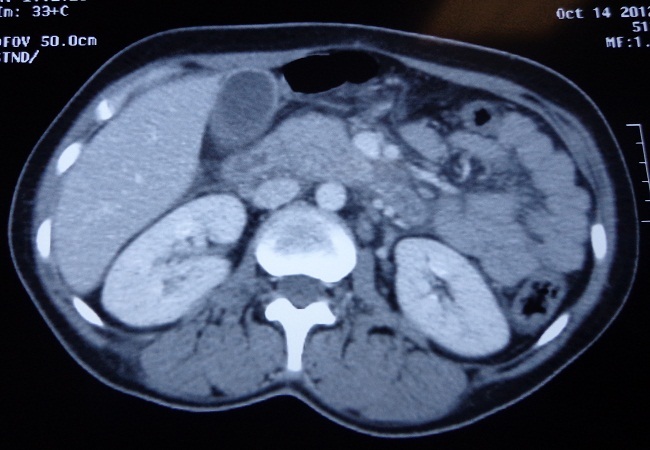
Pancréatite stade E sur le scanner abdominal

Mme H.A âgée de 24 ans, primigeste et primipare, sans antécédents pathologiques particuliers, est hospitalisée le 13 octobre 2012 au service de réanimation polyvalente du CHU Hassan II de Fès, pour la prise en charge des douleurs abdominales survenant 15 jours après un accouchement par voie basse. Le début de la symptomatologie remonte à trois jours avant son hospitalisation par l'apparition d'une manière brutale des douleurs épigastriques transfixiantes, irradiantes vers le dos, associées à des nausées et vomissements. L'examen à son admission a trouvé une patiente consciente, pâle, très algique, avec un BMI à 22 kg/m^2^, apyrétique avec une polypnée à 20 cycles/min et une tachycardie à 100 battements/min. La pression artérielle était à 100/60 mmHg. L'abdomen était souple avec une sensibilité épigastrique. Par ailleurs l'examen gynécologique, cardio-vasculaire et respiratoire était sans particularité. Les mollets sont souples. Le bilan biologique a révélé une Lipasémie élevée à 4601 UI/l, des GOT à 268 UI/l, GPT à 175 UI/l, CRP à 30mg/l, les globules blancs à 7980/mm^3^, une hémoglobine à 5.7 g/dl, plaquettes à 395000/mm^3^, taux de prothrombine à 87% et un TCA normal. La radiographie pulmonaire était normale. Le scanner abdominal a objectivé une pancréatite stade E selon la classification de Balthazar avec présence de plusieurs coulées de nécrose en péripancréatiques, hilaire splénique et au niveau des deux espaces para-rénaux antérieures (Figure 1) Pour la recherche étiologique, l'interrogatoire a écarté une prise médicamenteuse ou alcoolique, la grossesse et l'accouchement se sont déroulés sans anomalies, l'exploration échographique a montré une vésicule biliaire fine et lithiasique avec une voie biliaire principale libre. Le bilan lipidique et la calcémie était sans anomalie (calcémie à 94 mg/l, cholestérolémie à 1.58 g/l, triglycérides à 1.57 g/l, LDL à 0.95 g/l, HDL à 0.32 g/l). La patiente a bénéficié d'une transfusion sanguine par deux concentrés globulaires, d'une perfusion par sérum salé physiologique et glucosé, d'un traitement antalgique et d'une prophylaxie de la maladie thromboembolique avec bonne évolution. Elle fut transférée quatre jours après au service de chirurgie digestive puis sortie avec un rendez-vous pour cholécystectomie sous cœlioscopie.

## Discussion

La pancréatite aigüe est une inflammation aigue de la glande pancréatique, secondaire à une autodigestion du pancréas par activation intrapancréatique des enzymes digestives [1]. La forme grave se traduit par une défaillance multiviscérale survenant la première semaine de la maladie et/ou par l'apparition souvent plus tardive de complications locales dont les principales sont l'infection des tissus nécrosés, l'abcès pancréatique, l'hémorragie rétropéritonéale et le pseudokyste [2]. L'association de la pancréatite aigüe avec la grossesse est rare. Son incidence est de 1 pour 1000 à 3000 grossesses [3]. Le risque augmente avec l’âge gestationnel. Ainsi, l'incidence respective est de 19%, 26%, 55% durant le 1^er^, 2^ème^ et 3 ème trimestre. La pancréatite aigüe peut-être responsable d'une morbi-mortalité fœto-maternelle importante. La mortalité f'tale varie entre 0.57% et 4.7%, elle est liée à la prématurité et à l'acidose [3, 4]. La survenue de la pancréatite en post-partum est rare, comme c'est le cas de notre patiente qui l'a présentée deux semaines après l'accouchement. Ramin a rapporté un seul cas de pancréatite du post-partum sur 43 cas de pancréatite associée à la grossesse et Fukami a rapporté un cas survenant deux heures après l'accouchement [4, 5]. Les principales causes sont représentées par les lithiases des voies biliaires (56%) et les dyslipidémies (38%). La pré-éclampsie reste exceptionnelle. Bahloul a rapporté un cas de pancréatite aigue nécrotico-hémorragique secondaire à une hypertension artérielle gravidique d’évolution fatale [5, 6]. Sur le plan physiopathologique, la fréquence de la lithiase cholestérolique au cours de la grossesse est secondaire à une augmentation du volume et de la durée de la vidange vésiculaires par l'effet de la progestérone qui entraine une hypotonie des voies biliaires et une hypertonie du sphincter d'oddi. Les pancréatites secondaires à l'hypertriglycéridémie sont plus fréquentes au cours du 3^ème^ trimestre. Elles sont liées à une augmentation physiologique et progressive des lipides au cours de la grossesse. Au cours de la pré-éclampsie, la pancréatite est due à l'ischémie pancréatique en rapport avec la vasoconstriction et l'altération de la microvascularisation de l'organisme. Le pronostic des pancréatites est surtout lié à la précocité du diagnostic, qui repose sur des critères cliniques (douleurs épigastriques, nausées, vomissements), biologiques (amylasémie, Lipasémie) et radiologiques (échographie abdominale, scanner abdominal, IRM) [6, 7]. Les principes du traitement de la pancréatite du post-partum sont les mêmes qu'en dehors de la grossesse, à savoir, la mise à jeun, le support nutritionnel par alimentation entérale ou parentérale et le traitement de la douleur. En cas de surinfection des coulées de nécrose, une antibiothérapie avec un drainage percutané ou chirurgical sont nécessaires [8–10].

## Conclusion

La pancréatite aigüe du post-partum est une complication rare. Les principales causes sont dominées par l'origine lithiasique et dyslipidémique. Elle nécessite une prise en charge multidisciplinaire, comportant chirurgiens, gastro-entérologues, radiologues, obstétriciens et anesthésiste-réanimateurs. Le pronostic fœto-maternel a connu ces dernières années une très bonne amélioration devant la rapidité du diagnostic et de la qualité de la réanimation maternelle et néo-natale.
